# Supplementary material for: The Impact of Influencers on Cigar Promotions: A Content Analysis of Large Cigar and Swisher Sweets Videos on TikTok
Source: Int J Environ Res Public Health. 2022 Jun 9;19(12):7064. doi: 10.3390/ijerph19127064 (PMC9222503; doi:10.3390/ijerph19127064)
Supplement: Supplementary file 1 [file ijerph-19-07064-s001.zip › ijerph-1661752-supplementary.pdf]

Supplement for:

The Impact of Influencers on Cigar Promotions: A Content Analysis of Large Cigar and Swisher Sweets Videos on TikTok

This file includes:

Supplemental Table S1. Views of videos with different cigar-related hashtags on TikTok

Supplemental Table S2. Average followers and engagement with large cigar and Swisher Sweets TikTok Videos

Supplemental Table S3. Multiple comparisons of video features between large cigar and Swisher Sweets videos

Supplemental Table S4. Multiple comparisons of featured individual demographics for videos with people between large cigar and Swisher Sweets videos

Supplemental Table S5. Multiple comparisons of video content features between influencer and non-influencer videos within each hashtag category

Supplemental Table S6. Multiple comparisons of featured individual demographics for videos with recognizable individuals between influencer and non-influencer videos within each hashtag category

Supplemental Table S7. Multiple comparisons of content features between large cigar and Swisher Sweets influencers' videos

Supplemental Table S8. Multiple comparisons of featured individual demographics between large cigar and Swisher Sweets influencers' videos

Supplemental Table S9. Predicting likes and views of large cigar videos with video content features

Supplemental Table S10. Predicting shares of large cigar videos with video content features

Supplemental Table S11. Predicting likes and views of Swisher Sweets videos with video content features

Supplemental Table S12. Predicting shares of Swisher Sweets videos with video content features

Supplemental Table S13. Different cutoffs of follower count in large cigar and Swisher Sweets videos

Supplemental Table S14. Multiple comparisons of video features between large cigar and Swisher Sweets videos using the 90th percentile of followers to define influencers

Supplemental Table S15. Multiple comparisons of featured individual demographics for videos with people between large cigar and Swisher Sweets videos using the top 90th percentile of followers to define influencers

Supplemental Table S16. Multiple comparisons of featured individual demographics between large cigar and Swisher Sweets influencers' videos using the top 90th percentile of followers to define influencers

Supplemental Table S1. Views of videos with different cigar-related hashtags on TikTok (as of Sep 2020)

Hashtag Categories	Hashtags	Number of Views
Large Cigars	**#cigar**	**88.4M**
	**#cigars**	**54.6M**
	#traditionalcigar	Not Found
	#traditionalcigars	Not Found
	#largecigar	Not Found
	#largecigars	Not Found
LCCs	#littlecigar	57K
	#littlecigars	Not Found
	#cigarillo	65K
	#cigarillos	427K
Swisher Sweets	**#swisher**	**7.8M**
	**#swishers**	**4.9M**
	**#swishersweet**	**1.7M**
	**#swishersweets**	**1.6M**

* hashtags used for the data collection of the current study were bolded.

Supplemental Table S2. Average number of followers and engagement with large cigar and Swisher Sweets TikTok Videos

Cigar Type	User Type	Average Follower Counts	Average Views	Average Likes	Average Shares
Large Cigars	Influencers	73,410	88,749	4,455	41
	Non-influencers	2,369	8,718	304	7
Swisher Sweets	Influencers	34,232	45,625	4,962	116
	Non-influencers	296	2,434	150	10

Supplemental Table S3. Multiple comparisons of video features between relevant large cigar and Swisher Sweets videos.

	Large Cigar Videos* N = 1,333	Swisher Sweets Videos N = 367	Adjusted *P value*	OR (95% CI)
**Video Content Features**				
Product Promotion	60 (4.5%)	4 (1.1%)	**0.005**	0.23 (0.08, 0.65)
Marketing Events	7 (0.5%)	2 (0.5%)	0.963	1.04 (0.21, 5.02)
Purchasing behavior	27 (2.0%)	36 (9.8%)	**< 0.001**	5.26 (3.15, 8.78)
Product Review	180 (13.5%)	13 (3.5%)	**< 0.001**	0.24 (0.13, 0.42)
Smoking Cigars	789 (59.2%)	67 (18.2%)	**< 0.001**	0.15 (0.12, 0.21)
Prevention	6 (0.5%)	0	0.501	0.28 (0.02, 4.94)
Arts and Crafts	0	38 (10.4%)	**< 0.001**	312.57 (19.15, 5101.25)
Cannabis Use	19 (1.4%)	106 (28.9%)	**< 0.001**	54.82 (33.23, 90.44)
Dancing	6 (0.5%)	44 (12.0%)	**< 0.001**	30.13 (12.73, 71.31)
Smoke Tricks	16 (1.2%)	9 (2.5%)	0.164	2.07 (0.91, 4.72)
Cardi B Music	0	42 (11.4%)	--	--
Showing off Swisher Sweets packages	0	69 (18.8%)	--	--
Flavor	7 (0.5%)	179 (48.8%)	**< 0.001**	180.47 (83.47, 389.71)
Audio Health Warnings	3 (0.2%)	0	0.900	0.52 (0.03, 10.04)
Written Health Warnings	0	0	--	--

*Reference group. OR = odds ratio for the presence of coded video characteristics; CI = confidence intervals. Haldane-Anscombe correction was applied when observed value = 0 when calculating odds ratio.). *P* values were adjusted with Bonferroni correction methods. Content features: "Cardi B music" and "showing off Swisher Sweets" were excluded from the analysis because these two content features were unique to Swisher Sweets videos.

Supplemental Table S4. Multiple comparisons of featured individual demographics for videos with people between large cigar and Swisher Sweets videos.

	Large Cigar Videos* N = 945	Swisher Sweets Videos N = 247	Adjusted *P value*	OR (95% CI)
**Gender**				
Presence of Males	827 (87.5%)	139 (56.3%)	**< 0.001**	0.19 (0.13 0.25)
Presence of Females	206 (21.2%)	119 (48.2%)	**< 0.001**	3.34 (2.49, 4.47)
**Race**				
Black	143 (15.1%)	54 (21.9%)	**0.017**	1.57 (1.11, 2.23)
White	759 (80.3%)	165 (66.8%)	**< 0.001**	0.49 (0.36, 0.67)
Spanish/Hispanic	77 (8.1%)	30 (12.2%)	0.067	1.56 (0.10, 2.44)
Asian	32 (3.4%)	22 (8.9%)	**0.001**	2.65 (1.50, 4.69)
**Age**				
Presence of Young Individuals	33 (3.5%)	84 (34.0%)	**< 0.001**	14.24 (9.21, 22.02)

*Reference group. OR = odds ratio for the presence of coded video characteristics; CI = confidence intervals. Haldane-Anscombe correction was applied when observed value = 0 when calculating odds ratio. *P* values were adjusted with Bonferroni correction methods.

Supplemental Table S5. Multiple comparisons of video content features between influencer and non-influencer videos within each hashtag category

	Large Cigar Videos				Swisher Sweets Videos			
	**Non-influencers* N = 594**	**Influencers N = 739**	**Adjusted *P value***	**OR (95%CI)**	**Non-influencers* N = 176**	**Influencers N = 191**	**Adjusted *P value***	**OR (95%CI)**
**Video Content Features**								
Product Promotion	19 (3.2%)	41 (5.5%)	0.109	1.78 (1.02, 3.09)	2 (1.1%)	2 (1.0%)	0.963	0.92 (0.13, 6.61)
Marketing Events	2 (0.3%)	5 (0.7%)	0.703	2.02 (0.39, 10.43)	1 (0.5%)	1 (0.5%)	0.963	0.92 (0.06, 14.84)
Purchasing behavior	5 (0.8%)	22 (3.0%)	**0.025**	3.61 (1.32, 12.28)	2 (1.1%)	34 (17.2%)	< **0.001**	18.84 (4.45, 79.71)
Product Review	34 (5.7%)	146 (19.8%)	< **0.001**	4.05 (2.74, 5.99)	4 (2.2%)	9 (4.5%)	0.509	2.13 (0.64, 7.03)
Smoking Cigars	388 (65.3%)	401 (54.3%)	< **0.001**	0.63 (0.50, 0.79)	45 (24.7%)	22 (11.1%)	**0.004**	0.38 (0.22, 0.66)
Prevention	2 (0.3%)	4 (0.5%)	0.886	1.61 (0.29, 8.83)	0	0	-	-
Arts and Crafts	0	0	-	-	22 (12.1%)	16 (8.1%)	0.457	0.64 (0.32, 1.26)
Cannabis Use	7 (1.2%)	12 (1.6%)	0.703	1.38 (0.54, 3.54)	63 (34.6%)	43 (21.7%)	**0.020**	0.52 (0.33, 0.82)
Dancing	4 (0.7%)	2 (0.3%)	0.695	0.40 (0.07, 2.19)	9 (4.9%)	35 (17.7%)	< **0.001**	4.16 (1.94, 8.94)
Smoke Tricks	9 (1.5%)	7 (0.9%)	0.695	0.62 (0.20, 1.89)	9 (4.9%)	0	**0.016**	0.05 (0.00, 0.80)
Cardi B Music	0	0	-	-	20 (10.9%)	22 (11.1%)	0.963	1.02 (0.53, 1.93)
Showing packages	0	0	-	-	39 (21.4%)	30 (15.2%)	0.296	0.65 (0.39, 1.11)
Flavor	2 (0.3%)	5 (0.7%)	0.703	2.02 (0.39, 10.43)	74 (40.7%)	105 (53.0%)	**0.041**	1.68 (1.11, 2.54)
Audio Health Warnings	0	3 (0.4%)	0.578	5.65 (0.29, 109.61)	0	0	-	-
Written Health Warnings	0	0	--	--	0	0	-	-

*Reference group. OR = odds ratio for the presence of coded video characteristics; CI = confidence intervals. Haldane-Anscombe correction was applied when observed value = 0 when calculating odds ratio. *P* values were adjusted with Bonferroni correction methods

Supplemental Table S6. Multiple comparisons of featured individual demographics for videos with recognizable individuals between influencer and non-influencer videos within each hashtag category

	Large Cigar Videos				Swisher Sweets Videos			
	Non-influencers* N = 387	Influencers N = 558	Adjusted *P value*	OR (95%CI)	Non-influencers* N = 106	Influencers N = 141	Adjusted *P value*	OR (95%CI)
**Gender**								
Presence of Males	335 (86.6%)	492 (88.2%)	0.613	1.16 (0.78, 1.71)	61 (56.5%)	78 (55.3%)	0.826	0.91 (0.55, 1.52)
Presence of Females	84 (21.7%)	122 (21.9%)	0.954	1.01 (0.74, 1.39)	48 (44.4%)	71 (48.3%)	0.593	1.23 (0.74, 2.03)
**Race**								
Black	76 (19.6%)	67 (12.0%)	**0.006**	0.56 (0.39, 0.80)	31 (28.7%)	23 (15.7%)	0.053	0.47 (0.26, 0.87)
White	288 (74.4%)	471 (84.4%)	**0.001**	1.86 (1.35, 2.57)	64 (59.3%)	101 (68.7%)	0.149	1.66 (0.97, 2.83)
Spanish/Hispanic	36 (9.3%)	41 (7.3%)	0.472	0.77 (0.48, 1.23)	21 (19.4%)	9 (6.1%)	**0.009**	0.28 (0.12, 0.63)
Asian	20 (5.2%)	12 (2.2%)	**0.034**	0.40 (0.18, 0.88)	6 (5.6%)	15 (10.2%)	0.346	1.98 (0.74, 5.30)
**Age**								
Presence of Young Individuals	22 (5.7%)	11 (2.0%)	**0.009**	0.33 (0.14, 0.73)	22 (20.4%)	62 (42.2%)	**0.002**	2.30 (1.69, 5.33)

* Reference group. OR = odds ratio for the presence of coded video characteristics; CI = confidence intervals. Haldane-Anscombe correction was applied when observed value = 0 when calculating odds ratio. *P* values were adjusted with Bonferroni correction methods.

Supplemental Table S7. Multiple comparisons of content features between large cigar and Swisher Sweets influencers' videos

	Large Cigar Influencers' Videos* n = 739	Swisher Sweets Influencers' Videos n = 191	Adjusted *P value*	OR (95% CI)
**Video Content Features**				
Product Promotion	41 (5.5%)	2 (1.0%)	**0.020**	0.18 (0.04, 0.75)
Marketing Events	5 (0.7%)	1 (0.5%)	0.814	0.77 (0.09, 6.65)
Purchasing behavior	22 (3.0%)	34 (17.2%)	**< 0.001**	7.06 (4.02, 12.40)
Product Review	146 (19.8%)	9 (4.5%)	**< 0.001**	0.20 (0.10, 0.40)
Smoking Cigars	401 (54.3%)	22 (11.1%)	**< 0.001**	0.11 (0.07, 0.18)
Prevention	4 (0.5%)	0	0.805	0.43 (0.03, 7.96)
Arts and Crafts	0	16 (8.1%)	**< 0.001**	139.05 (8.30, 2229.00)
Cannabis Use	12 (1.6%)	43 (21.7%)	**< 0.001**	17.60 (9.06, 34.19)
Dancing	2 (0.3%)	35 (17.7%)	**< 0.001**	82.68 (19.68, 347.35)
Smoke Tricks	7 (0.9%)	0	0.482	0.26 (0.01, 4.48)
Flavor	5 (0.7%)	105 (53.0%)	**< 0.001**	179.23 (71.10, 451.80)
Audio Health Warnings	3 (0.4)	0	0.935	0.55 (0.03, 10.7)
Written Health Warnings	0	0	--	--

* Reference group. OR = odds ratio for the presence of coded video characteristics; CI = confidence intervals. Haldane-Anscombe correction was applied when observed value = 0 when calculating odds ratio.) *P* values were adjusted with Bonferroni correction methods.

Supplemental Table S8. Multiple comparisons of featured individual demographics between large cigar and Swisher Sweets influencers' videos

	Large Cigar Influencers' Videos* n = 558	Swisher Sweets Influencers' Videos n = 141	Adjusted *P value*	OR (95% CI)
**Gender**				
Presence of Males	492 (88.2%)	78 (53.1%)	**< 0.001**	0.17 (0.11, 0.25)
Presence of Females	122 (21.9%)	71 (48.3%)	**< 0.001**	3.62 (2.46, 5.33)
**Race**				
Black	67 (12.0%)	23 (15.7%)	0.258	1.43 (0.85, 2.39)
White	471 (84.4%)	101 (68.7%)	**0.001**	0.47 (0.30, 0.72)
Spanish/Hispanic	41 (7.3%)	9 (6.1%)	0.830	0.86 (0.41, 1.81)
Asian	12 (2.2%)	15 (10.2%)	**< 0.001**	5.42 (2.47, 11.86)
**Age**				
Presence of Young Individuals	11 (2.0%)	62 (42.2%)	**< 0.001**	39.03 (19.71, 77.29)

*Reference group. OR = odds ratio for the presence of coded video characteristics; CI = confidence intervals. Haldane-Anscombe correction was applied when observed value = 0 when calculating odds ratio. *P* values were adjusted with Bonferroni correction methods.

Modeling video engagement with video content Features

In order to identify the video content features that are associated with greater engagement of a cigar and Swisher Sweets video (i.e., number of views, likes and shares), we formulated negative binomial models for views and likes, and negative binomial hurdle models for shares within each of the cigar and Swisher Sweets datasets, respectively.

View and like counts in our dataset were over-dispersed (i.e., the distribution variance was greater than the mean). Negative binomial models can account for overdispersion in count data, which have been used in prior research on social media engagement (e.g., post 'likes'). The share counts in our dataset were not only over-dispersed, but also had excess zeros – more than 45% of all analyzed videos received zero shares. For the current study, we formulated a hurdle model to examine the effects of different video content features on numbers of shares. Hurdle models consist of two components: a binomial probability model that estimates the likelihood of observing at least one activity (in the current study, a video share), and a truncated count model to predict positive counts. Previous research has also used hurdle models to analyze social media engagement data (e.g., shares of Twitter posts).

Hurdle models are essentially two regression models that predict: 1) the zero-portion in the data and 2) the non-zero counts of the data. In the Hurdle models, the zero-portion model determines the probability of observing at least one activity (in the current study, a video share) with a binomial probability model. The non-zero portion model evaluates the probability of receiving positive counts with a zero-truncated negative binomial model. The exponentiated regression coefficients in the zero-portion model are treated as odds ratios (**OR**) and the exponentiated regression coefficients in the non-zero portion model are treated as incident rate ratios (**IRR**) when interpreting results.

The majority of our data were independent (85% of users had only one video in our dataset). However, there were some sources of non-independence due to the 15% of users who posted more than one video. Thus, we also included random effects of video authors in the negative binomial and Hurdle models. Variance inflation factor (**VIF**) scores for all independent variables were within the range of 0 to 2, indicating no substantive multicollinearity.

Supplemental Table S9. Predicting likes and views of large cigar videos with video content features for large cigar videos

	**Likes**		**Views**	
	Adjusted *P value*	IRR (CI)	Adjusted *P value*	IRR (CI)
Intercept	< **0.001**	187.45 (153.72, 228.59)	< **0.001**	3896.97 (3153.88, 4815.14)
Follower Count (Per 1000 followers)	< **0.001**	1.008 (1.006, 1.010)	< **0.001**	1.007 (1.005, 1.009)
Product Promotion	< **0.001**	0.30 (0.21, 0.44)	< **0.001**	0.32 (0.22, 0.47)
Purchasing behavior	< **0.001**	29.03 (17.03, 49.47)	< **0.001**	20.92 (11.98, 36.53)
Product Review	< **0.001**	2.50 (1.98, 3.17)	< **0.001**	2.03 (1.58, 2.61)
Smoking Cigars	< **0.001**	0.65 (0.54, 0.77)	< **0.001**	0.56 (0.46, 0.67)
Cannabis Use	0.900	1.05 (0.50, 2.17)	0.760	0.89 (0.43, 1.84)
Smoke Tricks	0.286	0.68 (0.35, 1.32)	0.457	0.74 (0.36, 1.50)

IRR (exponent of coefficient, Incident Rate Ratio); The models excluded content features including arts and crafts, Cardi B music, showing off Swisher Sweets packages, marketing events, prevention, written health warnings, audio health warnings and flavor for their rare occurrences.

Supplemental Table S10. Predicting shares of large cigar videos with video content features for large cigar videos

	Zero Portion		Non-zero Count Portion	
	Adjusted *P value*	OR (CI)	Adjusted *P value*	IRR (CI)
Intercept	0.482	1.18 (0.86, 1.60)	0.993	0.00 (0.00, Inf)
Follower Count (Per 1000 followers)	**< 0.001**	0.98 (0.97, 0.10)	**0.003**	1.01 (1.00, 1.02)
Product Promotion	0.299	0.53 (0.24, 1.16)	**0.019**	0.29 (0.12, 0.74)
Purchasing behavior	0.448	0.47 (0.14, 1.59)	**< 0.001**	44.70 (9.13, 218.86)
Product Review	0.299	0.64 (0.38, 1.07)	0.120	1.80 (0.94, 3.43)
Smoking Cigars	0.795	1.04 (0.76, 1.43)	**0.007**	0.47 (0.29, 0.77)
Cannabis Use	0.768	0.76 (0.21, 2.73)	0.958	1.22 (0.18, 8.06)
Smoke Tricks	0.768	0.71 (0.21, 2.39)	0.941	0.73 (0.14, 3.73)

Exponent of coefficient (OR, Odds Ration and IRR, Incident Rate Ratio). The models excluded content features including arts and crafts, Cardi B music, showing off Swisher Sweets packages, marketing events, prevention, written health warnings, audio health warnings and flavor for the reason of rare occurrences. The zero-portion model determines the probability of observing at least one activity (in the current study, a video share). The non-zero count portion model evaluates the probability of receiving positive counts.

Supplemental Table S11. Predicting likes and views of Swisher Sweets videos with video content features for Swisher Sweets videos

	Likes		Views	
	Adjusted *P value*	IRR (CI)	Adjusted *P value*	IRR (CI)
Intercept	< **0.001**	36.79 (22.57, 59.99)	< **0.001**	698.81 (431.06, 1132.86)
Follower Count	**0.001**	1.01 (1.00, 1.01)	**0.002**	1.01 (1.00, 1.01)
Purchasing behavior	0.126	2.19 (0.88, 5.43)	**0.036**	2.96 (1.26, 7.00)
Product Review	0.901	1.13 (0.39, 3.29)	0.853	1.10 (0.39, 3.10)
Smoking Cigars	**0.005**	0.37 (0.21, 0.68)	**0.001**	0.32 (0.18, 0.59)
Cannabis Use	0.096	1.59 (0.98, 2.58)	0.780	1.12 (0.70, 1.80)
Arts and Crafts	0.095	2.28 (1.03, 5.07)	0.124	2.13 (0.98, 4.63)
Showing Packages	0.573	0.79 (0.42, 1.50)	0.780	0.86 (0.46, 1.61)
Dancing	0.096	1.95 (0.97, 3.92)	0.780	1.25 (0.63, 2.47)
Cardi B Music	0.922	1.04 (0.49, 2.22)	0.780	0.87 (0.41, 1.82)
Flavored Products	0.079	1.76 (1.06, 2.92)	0.376	1.38 (0.84, 2.27)

IRR (exponent of coefficient, Incident Rate Ratio); The models excluded content features including prevention, written health warnings, audio health warnings, marketing events, and smoke tricks for the reason of rare occurrences.

Supplemental Table S12. Predicting shares of Swisher Sweets videos with video content features for Swisher Sweets videos

	Zero Portion		Non-zero Count Portion	
	Adjusted *P value*	OR (CI)	Adjusted *P value*	IRR (CI)
Intercept	**< 0.001**	5.26 (2.70, 10.24)	0.997	0.00 (0.00, Inf)
Follower Count	**< 0.001**	0.96 (0.94, 0.97)	0.758	1.00 (0.99, 1.01)
Purchasing behavior	0.848	0.85 (0.29, 2.50)	0.708	2.06 (0.16, 26.63)
Product Review	0.510	1.76 (0.45, 6.89)	0.708	3.20 (0.08, 134.81)
Smoking Cigars	0.863	0.94 (0.44, 1.99)	**0.008**	0.01 (0.00, 0.17)
Cannabis Use	0.374	0.66 (0.36, 1.20)	0.708	1.75 (0.33, 9.12)
Arts and Crafts	0.055	0.34 (0.14, 0.81)	0.708	3.48 (0.42, 28.59)
Showing Packages	0.510	0.71 (0.33, 1.53)	0.708	0.48 (0.07, 3.11)
Dancing	0.385	0.60 (0.27, 1.33)	0.708	2.43 (0.36, 16.57)
Cardi B Music	0.374	0.51 (0.21, 1.25)	0.269	0.04 (0.00, 0.98)
Flavored Products	0.453	0.71 (0.37, 1.34)	0.708	2.37 (0.45, 12.45)

Exponent of coefficient (OR, Odds Ration and IRR, Incident Rate Ratio). The models excluded content features including prevention, written health warnings, audio health warnings, marketing events, and smoke tricks for the reason of rare occurrences. The zero-portion model determines the probability of observing at least one activity (in the current study, a video share). The non-zero count portion model evaluates the probability of receiving positive counts

Sensitivity analysis for the influencer identification with 75th and 90th percentile of follower count

As detailed in the discussion section of the main text, one limitation of the paper is the method we used to identify influencers in the dataset. Specifically, we identified influencers as those in the top 75th percentile for the number of followers. Even though this method considers the distribution of follower counts in the large cigars and Swisher Sweet datasets, the selection of the 75th percentile was arbitrary. To address this limitation, we compared the findings of the content characteristics and individual features in the two types of cigar types, using two different percentiles (75 and 90) of followers to identify the influencers. Using the top 90th percentile of followers, we identified 61 large cigar influencers and 54 Swisher Sweets influencers (see details in **Supplemental Table 13**). The 61 large cigar influencers posted 350 videos and the 54 Swisher Sweets influencers posted 80 videos. Similar to the main study, we randomly sampled 350 large cigar videos from non-influencers in the large cigar dataset, and 80 Swisher Sweets videos from non-influencers in the Swisher Sweets dataset. The final sample size for the sensitivity analysis with the 90th percentile cutoff was 860 videos (700 large cigar videos and 160 Swisher Sweets videos).

A key finding of our study is that the demographics of the featured individuals and influencers, as well as content features differ between videos of large cigar and Swisher Sweets on TikTok. Hence, we performed the same Chi-square analyses to compare the individual demographics and content features in videos of the two cigar types using the 2 different percentile cutoffs to define the influencers. Similar to the main study, Chi-square analyses were conducted using SPSS (Version 26) with an alpha level of 0.05 (2-tailed) with Bonferroni correction to account for multiple testing. Chi-square analyses with the 90th percentile of followers showed similar findings as to the Chi-square analyses with the 75th percentile. Details of the results are shown below in **Supplemental Table 14, Supplemental Table 15, Supplemental Table 16.**

Supplemental Table S13. Identification of influencers using different cutoffs of follower count in large cigar and Swisher Sweets videos

		Follower count	Influencer count	Influencer post count
Large Cigar	75th percentile	15,000	190	877
	90th percentile	61,700	61	350
Swisher Sweets	75th percentile	1,759	147	226
	90th percentile	11,970	54	80

Supplemental Table S14. Multiple comparisons of video features between large cigar and Swisher Sweets videos using the 90th percentile of followers to define influencers

	Large Cigar Videos* N = 700	Swisher Sweets Videos N = 160	Adjusted *P value*	OR (95% CI)
**Video Content Features**				
Product Promotion	47 (6.7%)	2 (1.3%)	**0.012**	0.18 (0.04, 0.73)
Marketing Events	5 (0.7%)	1 (0.6%)	0.903	0.87 (0.10, 7.53)
Purchasing behavior	17 (2.4%)	28 (17.5%)	**< 0.001**	8.52 (4.54, 16.01)
Product Review	132 (18.9%)	6 (3.8%)	**< 0.001**	0.17 (0.08, 0.39)
Smoking Cigars	382 (54.6%)	26 (16.3%)	**< 0.001**	0.29 (0.19, 0.46)
Prevention	3 (0.4%)	0	0.407	0.62 (0.03, 12.08)
Arts and Crafts	0	12 (7.5%)	**< 0.001**	117.93 (5.93, 2002.95)
Cannabis Use	8 (1.1%)	44 (27.5%)	**< 0.001**	346.00 (132.43, 903.98)
Dancing	2 (0.3%)	18 (11.3%)	**< 0.001**	44.24 (10.14, 192.79)
Smoke Tricks	8 (1.1%)	4 (2.5%)	0.187	2.22 (0.66, 7.46)
Cardi B Music	0	22 (13.8%)	--	--
Showing off Swisher Sweets packages	0	27 (16.9%)	--	--
Flavor	5 (0.7%)	82 (51.2%)	< **0.001**	146.13 (57.49, 371.37)
Audio Health Warnings	3 (0.4%)	0	0.407	0.62 (0.03, 12.08)
Written Health Warnings	0	0	--	--

*Reference group. OR = odds ratio for the presence of coded video characteristics; CI = confidence intervals. Haldane-Anscombe correction was applied when observed value = 0 when calculating odds ratio.). *P* values were adjusted with Bonferroni correction methods. Content features: “Cardi B music” and “showing off Swisher Sweets” were excluded from the analysis because these two content features were unique to Swisher Sweets videos.

Supplemental Table S15. Multiple comparisons of featured individual demographics for videos with people between large cigar and Swisher Sweets videos using the top 90th percentile of followers to define influencers

	Large Cigar Videos* N = 524	Swisher Sweets Videos N = 111	Adjusted *P value*	OR (95% CI)
**Gender**				
Presence of Males	480 (91.6%)	65 (58.6%)	**< 0.001**	0.13 (0.08, 0.21)
Presence of Females	90 (17.2%)	49 (44.1%)	**< 0.001**	3.81 (2.46, 5.91)
**Race**				
Black	56 (10.7%)	24 (21.6%)	**0.014**	2.31 (1.35, 3.92)
White	445 (84.9%)	72 (64.9%)	**< 0.001**	0.33 (0.21, 0.52)
Spanish/Hispanic	42 (8.0%)	12 (10.8%)	0.337	1.39 (0.71, 2.74)
Asian	18 (3.4%)	13 (11.7%)	**< 0.001**	3.73 (1.77, 7.86)
**Age**				
Presence of Young Individuals	12 (2.3%)	40 (36%)	**< 0.001**	24.0 (12.0, 47.9)

*Reference group. OR = odds ratio for the presence of coded video characteristics; CI = confidence intervals. Haldane-Anscombe correction was applied when we observed value = 0 when calculating odds ratio. *P* values were adjusted with Bonferroni correction methods.

Supplemental Table S16. Multiple comparisons of featured individual demographics between large cigar and Swisher Sweets influencers' videos using the top 90th percentile of followers to define influencers

	Large Cigar Influencers' Videos* n = 285	Swisher Sweets Influencers' Videos n = 61	Adjusted *P value*	OR (95% CI)
**Gender**				
Presence of Males	274 (96.1%)	39 (63.9%)	**< 0.001**	
Presence of Females	42 (14.7%)	24 (39.3%)	**< 0.001**	
**Race**				
Black	11 (3.9%)	7 (11.5%)	0.105	3.23 (1.20, 8.70)
White	264 (92.6%)	42 (68.9%)	**< 0.001**	0.18 (0.09, 0.35)
Spanish/Hispanic	25 (8.8%)	5 (8.2%)	0.885	0.93 (0.34, 2.53)
Asian	4 (1.4%)	12 (19.7%)	**< 0.001**	17.2 (5.33, 55.52)
**Age**				
Presence of Young Individuals	2 (0.7%)	24 (39.3%)	**< 0.001**	91.78 (20.84, 404.27)

*Reference group. OR = odds ratio for the presence of coded video characteristics; CI = confidence intervals. Haldane-Anscombe correction was applied when observed value = 0 when calculating odds ratio. *P* values were adjusted with Bonferroni correction methods
